# An Explicit-Correction-Force Scheme of IB-LBM Based on Interpolated Particle Distribution Function

**DOI:** 10.3390/e25030526

**Published:** 2023-03-17

**Authors:** Bowen Liu, Weiping Shi

**Affiliations:** School of Mathematics, Jilin University, Changchun130012, China; liubowen0830@gmail.com

**Keywords:** lattice Boltzmann method, immersed boundary method, fluid–structure interaction, deformable body

## Abstract

In order to obtain a better numerical simulation method for fluid–structure interaction (FSI), the IB-LBM combining the lattice Boltzmann method (LBM) and immersed boundary method (IBM) has been studied more than a decade. For this purpose, an explicit correction force scheme of IB-LBM was proposed in this paper. Different from the current IB-LBMs, this paper introduced the particle distribution function to the interpolation process from the fluid grids to the immersed boundary at the mesoscopic level and directly applied the LBM force models to obtain the interface force with a simple form and explicit process. Then, in order to ensure the mass conservation in the local area of the interface, this paper corrected the obtained interface force with the correction matrix, forming the total explicit-correction-force (ECP) scheme of IB-LBM. The results of four numerical tests were used to verify the order of accuracy and effectiveness of the present method. The streamline penetration is limited and the numerical simulation with certain application significance is successful for complex boundary conditions such as the movable rigid bodies (free oscillation of the flapping foil) and flexible deformable bodies (free deformation of cylinders). In summary, we obtained a simple and alternative simulation method that can achieve good simulation results for engineering reference models with complex boundary problems.

## 1. Introduction

Fluid–structure interaction (FSI) has always been a research problem faced by various disciplines of natural science, such as the movement of fish and birds, the vibration of bridges, the computation of flow in porous media, the deformation of cells, the blood flow in the heart, the transportation of solid particles, and the surface design of cars and airplanes in aerodynamics. In the past few decades, researchers have made great efforts in the numerical simulation of FSI [1,2,3,4,5].

At present, the general method for complex fluid–solid coupling problems is the decoupling algorithm [6], which decouples and solves multiple equations involved in the problem. It has strong adaptability to complex problems and low computational complexity, and it is easy to integrate the existing advantages of solving single-type equations (such as fluid control equations and solid mechanics equations). Under the decoupling algorithm, the fluid–solid solution algorithm can be divided into two types according to the grid-processing technology of the fluid–solid interface: one is the body-fitted grid method, and the other is the fixed grid method. For the body-fitted grid method, the Arbitrary Lagrangian–Eulerian method is usually used [7,8]. This type of method has the advantage of ensuring a clear interface, but it has some drawbacks for some fluid problems. For example, when the grid deforms greatly, it is very difficult to construct a robust and high-accuracy scheme. Simultaneously, this method increases the computational cost due to the frequent mesh regeneration process, especially when considering complex and/or three-dimensional geometric shapes. To avoid these shortcomings, fixed grid technology uses regular fixed grids to discretize the fluid field, describing the influence of solids on the surrounding fluid with the additional physical force. This simplicity greatly reduces the computational cost and is very popular in FSI with complex geometries. However, the fixed grid method usually uses the interpolation method to process the interface, which will affect the accuracy of the numerical scheme. The development of a method that takes both speed and accuracy into consideration has never stopped in these decades. The immersive boundary method and the lattice Boltzmann method are typical representatives of fixed grid methods before they are combined.

The immersed boundary (IB) method was proposed by Peskin [9]: the flow field is solved on a fixed Euler grid, the interface is represented by Lagrangian grids, and the interface force which is treated as the source term of the fluid equation is obtained by the solid equation. This approach overcomes the defects of large deformation and is suitable for dealing with complex interface problems. The original IBM has first-order accuracy of space and the sharp boundary is smoothed to only one mesh width [10]. The substantial advantages of the IB method drive researchers to make further efforts to improve its accuracy. Yang, X. et al. [11] and Bao, Y. et al. [12] developed an optimization method of the interpolation functions. Fadlun et al. developed a ghost cell method [13]. Yang, J. [14] used the bi-linear interpolation scheme for IBM [14]. Griffith reviewed IBMS for elastic bodies and others [15]. The test proves the effectiveness of the IBMs for biological simulation.

LBM is a kind of computational fluid dynamics method for Euler grids [16,17]. This method can calculate flow fields under many complex conditions [18], such as turbulence for high Reynolds number problems, rarefied gas for big Knudsen numbers, thermal, sound waves, etc. Although most papers addressing IB-LBM only treat LBM as an alternative to the N-S solver, we think this underestimates the ability of LBM to solve the flow field. The LBM programming is simple and easy to parallelize, guaranteeing calculation speed [19]. The pure LBM method can also solve fluid–solid problems, and the difficulty lies in the treatment of boundary conditions. In past research, many boundary schemes of the complex boundary conditions have been developed, such as bounce-back interpolation scheme [20], non-equilibrium interpolation schemes [21], and so on. However, most of the LBM boundary conditions require the computation of the intersection of Euler mesh and solid boundary so far. Especially for moving rigid bodies by the LB model [22,23,24,25], the solid boundary, the internal solid nodes, and the external fluid nodes need to be constantly replaced, although some methods, such as the interpolation bounce method, can guarantee second-order accuracy [26,27].

The combination of IB-LBM is a manifestation of complementary advantages. The IBM of the IB-LBM can be regarded as a special LBM complex boundary treatment scheme, and its main advantage is the forward tracking feature, that is, the shape of the boundary is directly known and does not need to be reconstructed. The implementation of LBM is relatively simple, and its numerical cost is small. Currently, IB-LBM can be applied to many numerical simulations [26,27,28,29,30,31], and the theoretical improvement of IB-LBM has never stopped. Feng first presented the combination of the IBM and LBM to simulate the rigid particle–fluid interaction [32]. They computed the force of the fluid–solid interaction using a penalty method; the idea of the penalty method is to assume a slight change for each Lagrangian marker point on the immersed boundary, and then add a penalty force for each point to offset this change, so the points will be pulled towards to their reference position. This explicit IB-LBM is easy to compute and implement but shows unideal accuracy and stability of the results because of the limitation of artificial parameters and the non-strict satisfaction of the no-slip boundary condition [32]. To avoid the use of artificial parameters in the explicit IB-LBM, Feng et al. used the direct-force IBM to calculate the interface force. Although the IB-LBM method has improved efficiency and accuracy, the process is complicated because the force form which is obtained by solving the N-S equations is complex [33,34]. Wu proposed an implicit velocity correction method for IB-LBM [35]. The core idea of the method is to divide the flow field velocity into uncorrected velocity and corrected velocity, and the force between the fluid and solid interface is given by solving the corrected velocity of nodes on the immersed boundary. In the benchmark tests of the rigid body in fluid, the phenomenon of streamline penetration of the traditional IB-LBM is effectively avoided, indicating that the method can meet the non-slip boundary condition [36]. However, this method increases the complexity and instability of the calculation because this method adds an interpolation matrix inversion and implicit process of it at each time step [37]. Kang presented a multi-direct-forcing IB-LBM to decrease the computation cost of the implicit IB-LBM [38]. This method inherits the idea of velocity correction in the implicit IB-LBM but uses an iterative scheme instead of constructing and inverting a large matrix at each time step. However, the iteration only occurs on lattice nodes closed to the interface. This method may lead to a large number of iterative steps [38]. For this reason, T Seta proposed a non-iterative implicit IB-LBM method, but this method obviously can only provide relatively low accuracy and slightly different results compared with implicit and iterative schemes [39]. Wang developed an improved approximation method to combine the implicit IB-LBM method [40]. Gsell uses an analytical approximation of the non-reciprocity error to correct immersed boundary force and prevent boundary slip and flow penetration [41]. And an extremely simple force amplification technique proposed for IBM in the correction of boundary slip can be a reference for IB-LBM [42]. Afra, B. et al. developed a robust lattice spring model (LSM) with the spring tension in multiple directions, which could be an implicit scheme of IB-LBM for large deformation issues; this is an effective model and obtains good results in deformation simulation [43]. Additionally, some fluid control problems of flexible filaments with good simulation results have been studied by LSM [29,30].

We call the methods mentioned above macro-scale methods. This type of method models the fluid–structure interface only at the macroscopic scale, and the modeled physical quantities are also macroscopic physical quantities, so the combined meaning of IBM and LBM will be limited. To discover a deeper level of physical meaning and calculation methods, a few papers discussed IB-LBM on a mesoscopic scale. Niu first proposed an IB-LBM on the mesoscopic scale called the momentum-exchange-based IB-LBM [44]. The basic idea of this method is to interpolate the velocity distribution function of the LBM as a physical quantity of IBM and use the bounce-back scheme to treat the distribution function on the coupled interface, and then the interaction force on the interface can be calculated by the momentum exchange method. Hu proposed an iterative method to improve IB-LBM based on momentum exchange so that the original method can better meet the no-slip boundary conditions [45]. Yuan extended the method to the calculation of flexible bodies and the numerical results showed the adaptability of the method in the flexible body cases [46]. Tao presented an IB-LBM in which the no-slip boundary condition is implemented directly by correcting the distribution functions near the interface [47]. Wang used the half-bounce-back scheme to construct a boundary treatment method so that the IB-LBM on the mesoscopic scale can effectively treat the large curvature boundary conditions [48].

Regarding the accuracy order of IB-LBM, current reports are inconsistent; some papers report first-order [27,49] and some papers report second-order accuracy [35,50].

In order to find an alternative and optimal method, first of all, this paper tried to find a new method with deeper physical significance and an acceptable order of accuracy at the mesoscopic scale. Secondly, it absorbed the advantages of several previous methods, such as the original method with a simple form, and the implicit method for satisfaction of the no-slip boundary conditions, etc. Finally, it simulated the more complex working conditions (such as unsteady flow, movable rigid body, and flexible body) identically to the current explicit methods. Different from the current IB-LBMs, this paper used the particle distribution function (a mesoscopic physical quantity rather than the macroscopic physical quantity velocity in IBM) for the interpolation process, directly applied the LBM force models of the Euler grids to the Lagrangian grids, and obtained the interface force. In order to ensure the mass conservation in the local area of the interface, this paper further corrected the obtained interface force with the matrixes, forming an explicit correction force scheme of the IB-LBM proposed in this paper. From the analysis of the four groups of results, it can be seen that the method proposed in this paper is a feasible and effective method.

## 2. Related Work

- **Lattice Boltzmann Equation**

For the fluid domain, the applicable range of the continuum hypothesis is limited. In order to expand the description range of the flow field by the fluid-governing equations, the fluid domain can be described by continuous Boltzmann equations according to the gas kinetic theory. Since the collision integral term which closes the continuous Boltzmann equation is very complicated, it is necessary to simplify the collision integral term. Additionally, the Boltzmann-BGK equation based on the linear collision assumption is the most widely used at present [51], which is given by

(1) $$\frac{\partial f}{\partial t}+\xi \cdot \nabla_{x}f+a\cdot \nabla_{\xi }\cdot f=-\frac{1}{\tau_{c}}\left[f-f^{\left(eq\right)}\right]$$

where $f=f\left(x,\xi ,t\right)$ is the particle distribution function, x is the space displacement vector, ξ is the velocity vector, t is the time, $f^{\left(eq\right)}$ is the equilibrium distribution function, $\tau_{c}$ is the relaxation time, and $\frac{1}{\tau_{c}}\left[f-f^{\left(eq\right)}\right]$ is the BGK collision term.

The Lattice Boltzmann equation is a discrete equation of the continuous Boltzmann equation realized by a special difference scheme. As for the applicable range of the continuum hypothesis, the flow field can also be described by the macroscopic equations of fluid mechanics, such as the N-S equation, the convection–diffusion equation, etc. The corresponding LB model equations of these macroscopic equations can be achieved through multi-scale expansion techniques. Among the IB-LBM calculation examples, the LBGK equation is the most typical [52].

Let $\left\{c_{0},c_{1},\ldots ,c_{n}\right\},\;\;i=1,2,\ldots ,n$ be the discrete velocity space; then, there is a discrete velocity distribution function $f_{i}=f_{i}\left(x,c_{i},t\right)$. Substituting these into Equation (1) and discretizing it in time and space, the lattice Boltzmann equation with external force term is given by

(2) $$f_{i}\left(x+c_{i}\Delta t,t+\Delta t\right)-f_{i}\left(x,t\right)=-\frac{1}{\tau }\left(f_{i}\left(x,t\right)-f_{i}^{eq}\left(x,t\right)\right)+S_{i}$$

where $\tau =\tau_{c}/\Delta t$ is the dimensionless relaxation time, usually τ>0.5 can guarantee stability.

It is worth noting that Equation (2) has second-order accuracy in space time but most IBMs and IB-LBMs have first-order accuracy [19].

The $S_{i}$ of Equation (2) is the collision source term, and the discrete force scheme of Guo [53] is commonly used:

(3) $$S_{i}=\left(1-\frac{\Delta t}{2\tau }\right)w_{i}\left(\frac{c_{i}-U}{c_{s}^{2}}+\frac{\left(c_{i}\cdot U\right)\cdot c_{i}}{c_{s}^{4}}\right)\cdot F\left(X,t\right)$$

The DnQm model proposed by Qian [54] is the most commonly used discrete velocity scheme and the D2Q9 model was used in this paper. The discrete velocity space $c_{i}$ is expressed by

(4) $$c_{i}=\left\{\begin{array}{l} 0\;\;\;\;\;\;\;\;\;\;\;\;\;\;\;\;\;\;\;\;\;\;\;\;\;\;\;\;\;\;\;\;\;\;\;\;\;\;\;\;\;\;\;\;\;\;\;\;\;\;\;\;\;\;\;\;\;\;\;\;\;\;\;\;\;\;\;i=0\\ \left(\cos\left[\left(i-1\right)\frac{\pi }{2}\right],\;\sin\left[\left(i-1\right)\frac{\pi }{2}\right]\right)\frac{\Delta x}{\Delta t}\;\;\;\;\;\;\;\;i=1,2,3,4\\ \left(\cos\left[\left(2i-1\right)\frac{\pi }{4}\right],\;\sin\left[\left(2i-1\right)\frac{\pi }{4}\right]\right)\frac{\Delta x}{\Delta t}\;\;\;\;i=5,6,7,8 \end{array}\right.$$

The discrete equilibrium distribution function is given by

(5) $$f_{i}^{eq}\left(X,t\right)=\rho w_{i}\left[1+\frac{c_{i}.u}{c_{s}^{2}}+\frac{{\left(c_{i}.u\right)}^{2}}{2c_{s}^{4}}-\frac{u^{2}}{2c_{s}^{2}}\right]$$

where $c_{s}\;$is the lattice sound velocity and $w_{i}$ is the weight coefficient given by

(6) $$c_{s}=\frac{1}{\sqrt{3}}\frac{\Delta x}{\Delta t},\;\;\;\;\;\;w_{i}=\left\{\begin{array}{c} \frac{4}{9}\\ \frac{1}{9}\\ \frac{1}{36} \end{array}\right.\;\;\;\begin{array}{c} i=0\;\;\;\;\;\\ i=1\sim 4\\ i=5\sim 8 \end{array}$$

The dynamic viscosity in the LBM is given by

(7) $$\upsilon =c_{s}^{2}\left(\tau -0.5\right)\Delta t$$

The macro quantity velocity ρ and momentum ρu can be obtained by

(8) $$\rho =\overset{}{\underset{i}{\sum }}f_{i},\;\;\;\;\;\rho u=\overset{}{\underset{i}{\sum }}c_{i}f_{i}+\frac{f\Delta t}{2}$$

- **Immersed Boundary Method**

**Figure 1:**
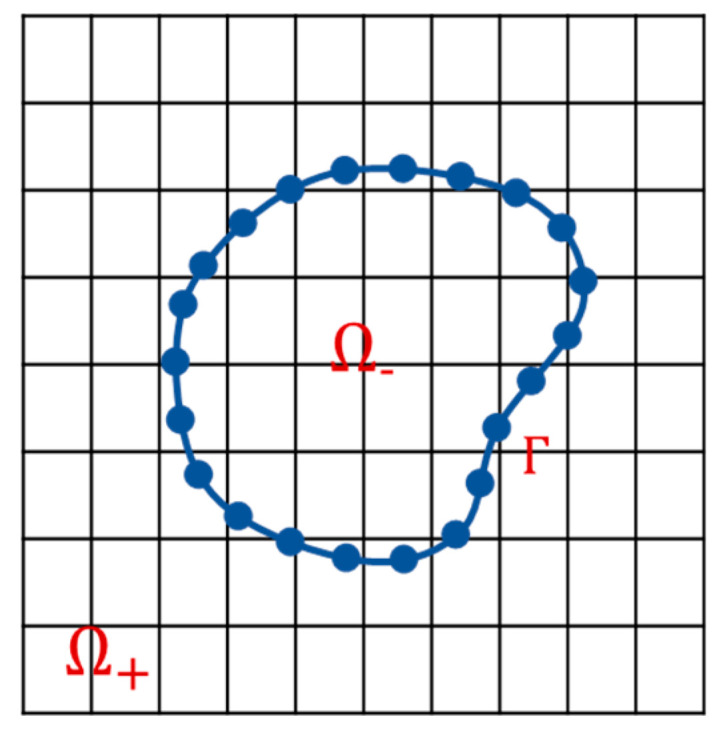
Computational domain with an immersed boundary in 2D.

As shown in Figure 1, IBM adopts the Euler–Lagrangian grid scheme. Consider a bounded region $\Omega \subset R^{n}\left(n=2,3\right)$ as the coupling region, $\Omega_{+}$ and $\Omega_{-}$ are the fluid and solid geometric regions respectively before immersion. Based on the immersed boundary assumption, let Γ=$\Omega_{+}\cap \Omega_{-}$ be the fluid–solid coupling-immersed interface, Ω=$\Omega_{+}\cup \Omega_{-}$ is the fluid calculation domain, and Γ satisfies the non-slip boundary condition.

The Euler coordinate points $x=\left(x_{i},i=1,2,3\right)\in \Omega$ describe the fixed grid coordinates of the fluid, and arc length coordinates $s\left(s_{i},i=1,2,3\right)\subset K$ as the Lagrange coordinates to describe interface particles. A mapping $X\left(s,t\right)\in \Omega$ is established to show the physical position of the particles with arc length parameters s at time t. Let L be the geometric length of the interface, K be the collection of particles, and $\Gamma_{t}$ be the shape of the interface at time t:

(9) $$\Gamma_{t}=X\left(K,t\right),\;\;X\left(0,t\right)=X\left(L,t\right)$$

For the interpolation of physical quantities, the delta function interpolation scheme proposed by Peskin [55,56] is the commonly used:

(10) $$f\left(x\right)=\int_{\Gamma }F\left(s\right)\delta \left(x-X\right)ds,\;U\left(X\right)=\int_{\Omega }u\left(x\right)\delta \left(x-X\right)dx$$

where $\delta \left(x\right)$ is the delta function, which can only guarantee first-order accuracy. f,u are force and velocity on the Euler grids for the fluid, and F, U are force and velocity on the Lagrangian grids for the solid. Additionally, F is determined by the constitutive relation at the immersed boundary, usually Hooke’s law.

As the comprehensive consideration of speed and error, two commonly used delta functions $\delta \left(x\right)$ [11] in this paper are given by

(11) $$\delta \left(x\right)=\left\{\begin{array}{l} \frac{1}{8}\left(3-2\left|x\right|+\sqrt{1+4\left|x\right|-4x^{2}}\right)\;\;\;\;\;\;\;\;\;\;\;\;\;\;\;\;\;\;\;\;\;\;\;\;(\left|x\right|<1)\\ \frac{1}{8}\left(5-2\left|x\right|-\sqrt{-7+12\left|x\right|-4x^{2}}\right)\;\;\;\;\;\;\;\;\;\;\;\;\;\;\;\;\;\;\left(1\leq \left|x\right|\leq 2\right)\\ 0\;\;\;\;\;\;\;\;\;\;\;\;\;\;\;\;\;\;\;\;\;\;\;\;\;\;\;\;\;\;\;\;\;\;\;\;\;\;\;\;\;\;\;\;\;\;\;\;\;\;\;\;\;\;\;\;\;\;\;\;\;\;\;\;\;\;\;\;\;\;\;\;\;\;\;\;\;(\left|x\right|>2) \end{array}\right.$$

A smooth function of $\delta \left(x\right)$ is given by

(12) $$\delta^{\prime }\left(x\right)=\left\{\begin{array}{l} \frac{1}{4}\left(1+\cos(\frac{\pi x}{2})\right)\;\;\;\;\;\;\;\;\;\;\;\;\;\;\;\;\left(\left|x\right|\leq 2\right)\\ 0\;\;\;\;\;\;\;\;\;\;\;\;\;\;\;\;\;\;\;\;\;\;\;\;\;\;\;\;\;\;\;\;\;\;\;\;\;\;\;\;\;\;(\left|x\right|>2) \end{array}\right.$$

- **Lattice Boltzmann-Immersed Boundary Method in other Work**

A traditional IB-LBM equation can be established through Equation (2), Equation (8), and Equation (10), but f,F, U are unknown. Once f or F is obtained, then U can be solved by solid equations.

We enumerate four representative IB-LBM methods at present. They are the traditional explicit method, the traditional implicit method, the direct stress integration method, and the bounce-back momentum exchange method, where the bounce-back momentum exchange method is a pioneering representative method of the mesoscopic method. The key to all methods is to obtain the f or F to solve the IB-LBM equations.

(1) The traditional explicit method [22]

The traditional explicit method is carried out under the assumption that the movement of the solid point satisfies Hooke’s law.

(13) F=k⋅ΔX

where ΔX is the displacement of the boundary Lagrangian point, F is the imaginary bound-back force due to ΔX, and k is the given stiffness parameter.

(2) The traditional implicit method [35]

The velocity correction method was proposed by Wu by solving a system of linear equations (see [35] for details); the correction velocity at the Euler point is implicitly obtained, and then the force density at the Euler node is obtained.

(14) $$f=2\rho \frac{u-\bar{u}}{\Delta t}=2\rho \frac{\Delta u}{\Delta t}$$

where $\bar{u}$ is the uncorrected velocity in fluid domain.

(3) The direct stress integration method [33]

In this method, the interface stress is obtained by introducing the N-S equation into the interface, which is similar to the stress integration method in the traditional LBM. The solution result of the solid force density is given by

(15) $$F_{\alpha }=\rho \frac{U_{\alpha }-u_{\alpha }}{\Delta t}+\rho u_{\beta }\partial_{\beta }u_{\alpha }+\partial_{\alpha }p-\mu \partial_{\beta }^{2}u_{\alpha }$$

where $p=c_{s}^{2}\rho$ is the positive pressure and α,β respectively represent the coordinate directions under the 2D condition of F.

(4) The bounce-back momentum exchange method [44]

The bounce-back momentum exchange method opens a precedent for exploring the mesoscopic immersed boundary method. This method is established under the condition of elastic collision. The interface force density is given by

(16) $$F=\overset{}{\underset{i}{\sum }}c_{i}\left(f_{i}^{new}-f_{i}\right)=\overset{}{\underset{i}{\sum }}c_{i}\left(f_{-i}-f_{i}-2w_{i}\rho \frac{c_{i}u}{c_{s}^{2}}\right)$$

where −i is the opposite direction of i and $f_{i}^{new}$ is the distribution function by the bound-back scheme.

## 3. The Present Explicit Correction Force Scheme for IB-LBM

When applying the direct stress integration method and the bound-back momentum exchange algorithm to solve the interface force, the problems of low accuracy and not satisfying the non-slip boundary are obvious [17]. For the former, it is easy to produce large noise, and the calculation is too cumbersome. This paper proposed a mesoscopic force calculation method that has a simple form and satisfies no-slip boundary conditions.

Based on the continuous medium around the interface and the physical quantity of the flow field being a continuous real number [57], we made the following assumptions:

(1) The discrete-velocity distribution function $f_{i}$ can be used as an interpolation physical quantity in the direction from Euler to Lagrangian in Equation (17). (The crash of our program may suggest that it cannot be used as an interpolation quantity from Lagrangian to Euler);
(2) The velocity U obtained from the solid Equations (21,22) on the Lagrangian point can be regarded as the equilibrium velocity of the LBM force model in Equation (8);
(3) The force model of LBM in Equation (8) is still applicable at the Lagrangian point.

Satisfying the three abovementioned assumptions, we derived the force equation on the immersed boundary, as follows.

- **Explicit Force by the Interpolated Distribution Function and LBM Force Models**

Owing to our assumptions, we can remove the limit of original fixed grid technology by the interpolation method of the velocity distribution function [58].

We defined the set of all Lagrangian marked points by $L_{p}=\left\{X_{1},X_{2},\cdots ,X_{N}\right\}$. For any $X_{n}\in L_{p}$, let $f_{i}\left(X_{n},t\right)$ be the reconstructed velocity distribution function at the Lagrangian point $X_{n}$. We have

(17) $$f_{i}\left(X_{n},t\right)=\overset{M}{\underset{m=1}{\sum }}f_{i}\left(x_{m},t\right)I\left(x_{m}-X_{n}\right)h^{d}$$

where h is the Euler grid length, d is the space dimension, and $I\left(x_{m}-X_{n}\right)$ is the discrete delta function [55]. Let $x_{m}\in E_{p}=\left\{x_{1},x_{2},\cdots ,x_{M}\right\}$ be the Euler coordinate point set to be interpolated. Additionally, abbreviate $I\left(x_{m}-X_{n}\right)$ as

(18) $$I_{mn}=I\left(x_{m}-X_{n}\right)=\frac{1}{h^{d}}\overset{d}{\underset{j=1}{\prod }}\delta \left(\frac{x_{m,j}-X_{n,j}}{h}\right)$$

where $x_{m,j}$ and $X_{n,j}$ are the coordinate values of the points $x_{m}$ and $X_{n}$ at the coordinate direction of j, respectively. $\delta \left(x\right)$ is given by Equation (11) or (12).

Under our assumptions, the moment equations satisfy

(19) $$\rho \left(X_{n},t\right)U\left(X_{n},t\right)=\overset{}{\underset{i}{\sum }}c_{i}f_{i}\left(X_{n},t\right)+AF\left(X_{n},t\right)\Delta t$$

where ρ and U are the density and velocity of the fluid at the Lagrangian point, respectively.

The selection of A is related to the choice of the LBM force model. We obtain get a conclusion from ref. [17]. A can be 0.5, τ, and 0 with different $S_{i}$ in Equation (2).

From Equations (19) and (20), the interfacial force exerted on the fluid by the solid can be calculated explicitly by

(20) $$F\left(X_{n},t\right)=\frac{\rho \left(X_{n},t\right)U\left(X_{n},t\right)-\sum_{i}c_{i}f_{i}\left(X_{n},t\right)}{A\Delta t}$$

The calculation of $U\left(X_{n},t\right)$ is determined by the equations of the solid. We provide a general calculation equation; for any $X_{n}\in L_{p}$, we have

(21) $$\rho_{s}\frac{\partial^{2}X}{\partial t^{2}}=f+F_{in}+F_{ext}$$

(22) $$U\left(X_{n},t\right)=\frac{\partial X}{\partial t}$$

where $\rho_{s}$ is the mass density of the solid node on the immersed boundary, f is the interface force exerted on the solid by the fluid, $F_{in}$ is the internal force generated inside the solid because of external forces, and $F_{ext}$ is the resultant force generated in other situations.

When the solid is a rigid body, we have

(23) $$U\left(X_{n},t\right)=0;$$

When the solid is a deformable body, we need further modeling in $F_{in}$ and $F_{ext}$ to obtain $U\left(X_{n},t\right)$; Section 4.4 shows an example for deformation.

If the $U\left(X_{n},t\right)$ is obtained, $F\left(X_{n},t+\Delta t\right)$ can be obtained.

- **Theoretical Correction of Explicit Force by Correction Matrix**

Because of the special nature of the δ function, the implicit method used the corrected velocity to obtain the velocity on the Euler grids [35]. Different from the implicit method obtaining the correct velocity on Euler grids, we directly corrected the force on the immersed boundary.

We need to modify the force $F\left(X_{n},t\right)$ obtained in Equation (20). Additionally, let $\bar{L}={\left[\bar{F}\left(X_{1},t\right),\bar{F}\left(X_{2},t\right),\ldots ,\bar{F}\left(X_{N},t\right)\right]}^{T}$ be the force density vector to be corrected at the Lagrangian point, $L={\left[F\left(X_{1},t\right),F\left(X_{2},t\right),\ldots ,F\left(X_{N},t\right)\right]}^{T}$ be the corrected force density vector at the Lagrangian point, and $E={\left[f\left(x_{1},t\right),f\left(x_{2},t\right),\ldots ,f\left(x_{M},t\right)\right]}^{T}$ be the force density vector on the Euler point.

The physical quantities spread from the Lagrangian point to the Euler point, satisfying the following spread discrete function which is similar as Equation (18):

(24) $$S_{mn}=S\left(x_{m}-X_{n}\right)=\frac{1}{h^{d}}\overset{d}{\underset{j=1}{\prod }}\delta \left(\frac{x_{m,j}-X_{n,j}}{h}\right)$$

IBM’s interpolation process introduces errors in the force calculations [35]. We think that the correct force is numerically lost in the process of interpolation and spreading, so we restored the current force to obtain the correct force from an inverse process.

Introducing spread function matrix S, interpolation function matrix I, Euler space unit matrix $E_{h}$, and Lagrangian space unit matrix $L_{s}$, we have

(25) $$IE_{h}E=\bar{L}$$

(26) $$SL_{s}L=E$$

where

(27) $$S=\left[\begin{array}{cccc} S_{11} & S_{12} & \cdots & S_{1N}\\ S_{21} & S_{22} & \cdots & S_{2N}\\ \cdots & \cdots & \ddots & \vdots \\ S_{M1} & S_{M2} & \cdots & S_{MN} \end{array}\right],\;\;\;\;I=\left[\begin{array}{cccc} I_{11} & I_{12} & \cdots & I_{1M}\\ I_{21} & I_{22} & \cdots & I_{2M}\\ \cdots & \cdots & \ddots & \vdots \\ I_{N1} & I_{N2} & \cdots & I_{NM} \end{array}\right]$$

(28) $$E_{h}=\left[\begin{array}{cccc} h^{d} & 0 & \cdots & 0\\ 0 & h^{d} & \cdots & 0\\ \cdots & \cdots & \ddots & \vdots \\ 0 & 0 & \cdots & h^{d} \end{array}\right],L_{s}=\left[\begin{array}{cccc} \Delta s_{1} & 0 & \cdots & 0\\ 0 & \Delta s_{2} & \cdots & 0\\ \cdots & \cdots & \ddots & \vdots \\ 0 & 0 & \cdots & \Delta s_{N} \end{array}\right]$$

After finishing Equations (25) and (26), we have

(29) $$IE_{h}SL_{s}L=\bar{L}$$

where $T=IE_{h}SL_{s}$ is the modified transformation matrix, then

(30) $$L=T^{-1}\bar{L}$$

So far, we have given a calculation method of correction force with matrix inversion. Obviously the matrix is a sparse matrix. The LU decomposition is suggested to be applied into Equation (30).

- **The Total Process for FSI by the Proposed Method**

Combining Equation (2), Equation (20), and Equation (30), we can obtain the correction force calculation difference equations proposed in this paper:

(31) $$f_{i}\left(x+c_{i}\Delta t,t+\Delta t\right)-f_{i}\left(x,t\right)=\sum \Omega_{i\;}\;on\;\Omega_{+}\cup \Omega_{-}\;$$

(32) $$L^{t}={\left(T^{t}\right)}^{-1}{\bar{L}}^{t}\;\;on\;\;\Gamma \;\;\;$$

(33) $$\rho_{s}\frac{U_{n}^{t+1}-U_{n}^{t}}{\Delta t}=f_{n}^{t}+F_{in,n}^{t}+F_{ext,n}^{t}\;\;on\;\;\Gamma$$

where $\Omega_{i}$ are the source term operators including the BGK collision operator and $L^{t}$ represents the vector of the force density on the Lagrangian point with a time step of t, that is, $L^{t}={\left[F_{1}^{t},F_{2}^{t},\ldots ,F_{N}^{t}\right]}^{T}$;similarly, we have ${\bar{L}}^{t},S^{t},I^{t},L_{s}^{t},E_{h}^{t}$ and $T^{t}$.

**Figure 2:**
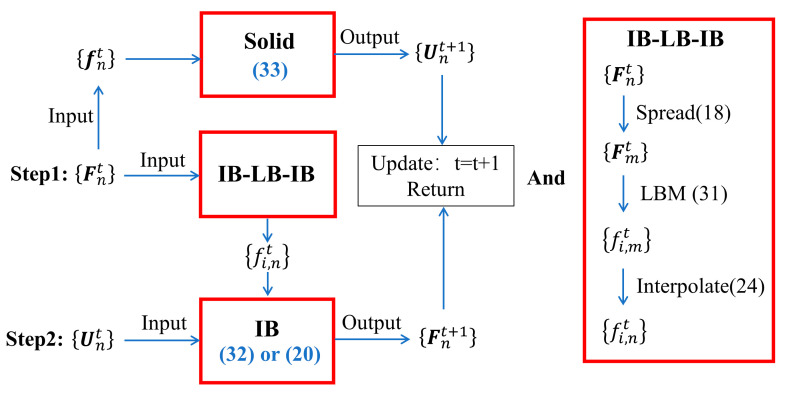
Flowchart of the new IB-LBM based on the proposed explicit correction force scheme.

Compared with the traditional explicit calculation method through the iterative program, it can be seen that the present method can realize the parallel calculation of fluids and solids. For the t-th time step, the following iteration is designed in Figure 2, where $\left\{P\right\}$ is the collection of physical quantity P at discrete points.

**Step 1**.

IB-LB-IB part in Figure 2:

Input $\left\{F_{n}^{t}\right\}$, and then the interpolated solid force density acting on the Euler point $\left\{f_{m}^{t}\right\}=\left\{\sum_{n=1}^{N}F_{n}^{t}S_{mn}\Delta s_{n}\right\}$; execute the LBM fluid solver (31) and output the Euler point velocity distribution function $\left\{f_{i,m}^{t}\right\}$ and the Lagrangian point velocity distribution function $\left\{f_{i,n}^{t}\right\}=\left\{\sum_{m=1}^{M}f_{i,m}^{t}I_{mn}h^{d}\right\}$. Then, update the macroscopic information of the flow field $\rho^{t}$, $u^{t}$.,

Solid part in Figure 2:

Input $\left\{F_{n}^{t}\right\}$, and then the fluid force density acting on the Lagrangian point $f_{n}^{t}=-F_{n}^{t}$; execute the solid solver (33) and output the velocity of the Lagrangian point $\left\{U_{n}^{t+1}\right\}$. Update the position of the immersed boundary $\left\{X_{n}^{t+1}\right\}$ at the same time.

**Step 2**.

IB part in Figure 2:

Input the $\left\{f_{i,n}^{t}\right\}$ of Step 1 and the $\left\{U_{n}^{t}\right\}$, and then execute the explicit correction force program (32) or explicit force program (20), and output $\left\{F_{n}^{t+1}\right\}$. Update *t* = *t* + 1 and return to Step 1.

For steady flows, set the threshold ε if the termination condition is met:

(34) $$\frac{{\left\|u\left(x,t\right)-u\left(x,t-\Delta t\right)\right\|}_{\infty }}{{\|u\left(x,t\right)\|}_{\infty }}<\varepsilon$$

For For unsteady flows, set the maximum simulation time $T=t_{max}$.

## 4. Results

### 4.1. Plane Poiseuille Flow

Poiseuille flow widely exists in industrial production and medical research and has guiding significance for the study of more complex physical problems [59].

In the following, a numerical experiment of plane Poiseuille flow is designed to verify the error and accuracy of the method proposed in this paper. The plane Poiseuille flow has the analytic solution in the velocity field which is expressed by

(35) $$u\left(y\right)=\frac{\Delta P}{\nu }\frac{D^{2}}{2}\left(\frac{y}{D}-\frac{y^{2}}{D^{2}}\right)$$

where y is the height coordinate in the channel, D is the width of the channel, ΔP is the pressure difference inlet and outlet, and ν is the dynamic viscosity.

The maximum velocity value $u_{max}$ is located at the center of the channel, which is calculated by

(36) $$u_{max}=\frac{\Delta PD^{2}}{8\nu }$$

**Figure 3:**
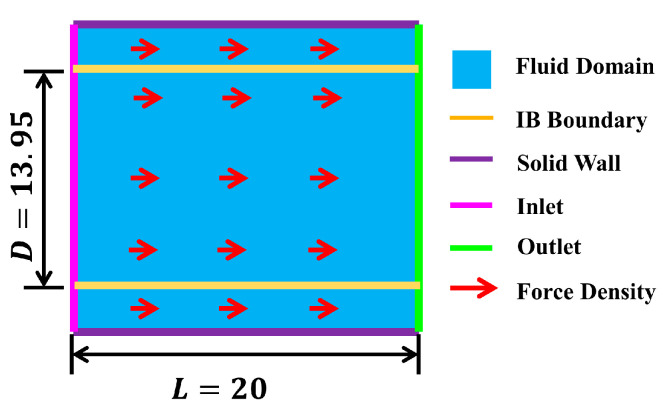
The design of the computational domain in 2D Poiseuille flow.

The design of the computational domain is shown in Figure 3, and the relevant parameters are dimensionless. Considering the Poiseuille flow in a rectangular domain, $\Omega =\left[0,L\right]\times \left[0,L\right]$, where L=20Δx and lattice length Δx=1, the left side is the inlet and the right side is the outlet, which are set as the periodic boundary. The upper and lower sides are fixed solid-wall boundaries, which are treated by the non-equilibrium scheme. Two rigid IB boundaries with distances of D=13.95 are set in the direction of the X axis. The force density of the Poiseuille flow is $\Delta P=1e^{-5}$. The Reynolds number Re=10 and the relaxation time τ=1 remain constant. The initial numbers of Euler grids Nx=20 in the X directions,Ny=20 in the Y directions. The initial number of Lagrangian grids on the IB is 18 and the grid ratio is about 1.17 and the arc lengths between grids are equal. The grid numbers of four tests in each group were increased by two times compared with the previous group, namely, 20×20, 40×40, 80×80, 160×160. The number of Lagrangian grids per group was also increased by the same multiple.

Two error norms are used in the error calculation of the velocity field. The infinite norm of the error is expressed by

(37) $${\left\|u^{n}-u^{a}\right\|}_{\infty }=\underset{\begin{array}{l} 1\leq i\leq Nx\\ 1\leq j\leq Ny \end{array}}{max}\left|u_{ij}^{n}-u_{ij}^{a}\right|$$

where $u_{ij}^{n},u_{ij}^{a}$ are the velocity numerical solution and the velocity analytical solution on the Euler grids, respectively.

The $L_{2}$ norm of the velocity error is given by

(38) $$\|u^{n}-u^{a}\|=\sqrt{\frac{\sum_{i=1}^{Nx}\sum_{j=1}^{Ny}{\left(u_{ij}^{n}-u_{ij}^{a}\right)}^{2}}{Nx\times Ny}}$$

**Figure 4:**
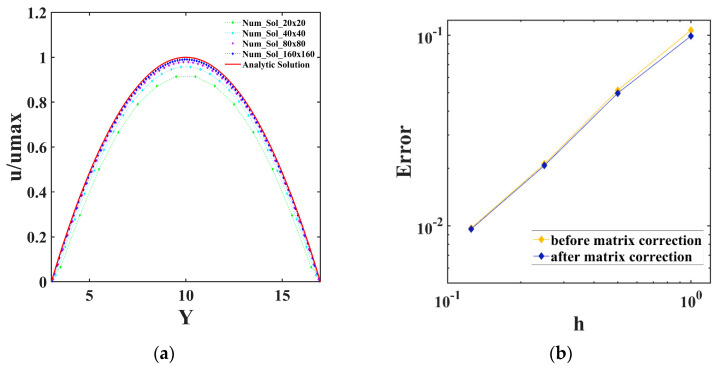
The numerical solutions of four cases: (**a**) compared with the analytic solution; (**b**) errors in the logarithmic scale of four cases before and after matrix correction.

Figure 4a is a comparison curve between the analytical solution and the numerical solution under different grid schemes in the plane Poiseuille flow. It can be seen that each numerical solution curve has a relatively high degree of fit with the analytical solution curve, which proves the effectiveness of the algorithm proposed in this paper under steady conditions. Figure 4b shows two error curves before and after matrix correction with the change of the space step. This shows that whether the matrix correction is added or not, the convergence order of the method proposed in this paper will not be changed. Additionally, the purpose of the matrix correction is to ensure the mass conservation of the interface part (Section 4.2).

Table 1 shows the specific results of the L2 norm and the infinite norm of the error under the four grid schemes. We see that the method proposed in this paper had first-order accuracy and the numerical results were consistent with the velocity analytical solution. Although LBM equations can obtain approximate second-order accuracy in space, most of the boundary treatments, such as elastic boundaries, are reduced to first-order accuracy.

As the computational advantage of the IB-LBMs is the excellent parallel strategy and it is reported that IB-LBMs are 23 times faster than the IBM Navier–Stokes solver [60] (though this is controversial), this explicit method can be better than IBM with the N-S solver and implicit IB-LBMs.

### 4.2. Flow over a Fixed Circular Cylinder

Determining flow over a circular cylinder is a classic test in fluid mechanics. A lot of experimental results and numerical results are available, so they are used to validate the IB-LBMs [38,39,40,41,42,43,44,45,46,47]. Herein, we further verify the accuracy of the IB-LBM proposed in this paper under the unsteady condition and mass conservation (the strictly satisfied no-slip boundary condition).

In this case, Re=U∞Dν, where $U_{\infty }$ is the inlet velocity, D is the diameter of the cylinder, and ν is the dynamic viscosity. The drag coefficient $C_{D}$ and the lift coefficient $C_{L}$ are obtained by

(39) $$C_{D}=\frac{F_{D}}{\frac{1}{2}\rho U_{\infty }^{2}D}$$

(40) $$C_{L}=\frac{F_{L}}{\frac{1}{2}\rho U_{\infty }^{2}D}$$

where the drag force $F_{D}=-\int_{\Omega }f_{x}dxdy=\int_{\Gamma }F_{x}ds$ and the lift force $F_{L}=-\int_{\Omega }f_{y}dxdy=\int_{\Gamma }F_{y}ds$; here, the subscripts x and y are the x-direction and y-direction in the domain.

The Strouhal number $S_{t}$ is defined to characterize the periodic effect in unsteady flow and expressed by

(41) $$S_{t}=\frac{f_{q}D}{U_{\infty }}$$

where $f_{q}$ is the vortex shedding frequency.

The whole computational domain considers a rectangular domain $\Omega =\left[0,400\Delta x\right]\times \left[0,600\Delta x\right]$, the lattice length Δx=1, the cylinder diameter D=32Δx, the number of the Lagrangian grids is 72, and the grid ratio is about 1.4. The upper and lower boundaries are fixed solid boundaries, the left is the inlet with the velocity $U_{\infty }=0.2$, and the right is the outlet with the velocity gradient of zero. The non-equilibrium scheme is used to deal with the abovementioned four boundaries [61]. The Reynolds numbers were set as 20, 40, 100, and 200 respectively, and the comparison of matrix correction (30) was also considered in the numerical simulations.

Table 2 shows the calculation and comparison results of three important parameters, i.e., drag coefficient $C_{D}$, lift coefficient $C_{L}$, and Strouhal number $S_{t}$. They were compared with the results of the experiment [62], other numerical methods [63], and other IB-LBMs [35,45]. The results showed that the numerical method proposed in this paper was almost identical to other results. This shows that the calculation accuracy of the method proposed in this paper is good and the numerical results under unsteady flow are therefore verified.

**Figure 5:**
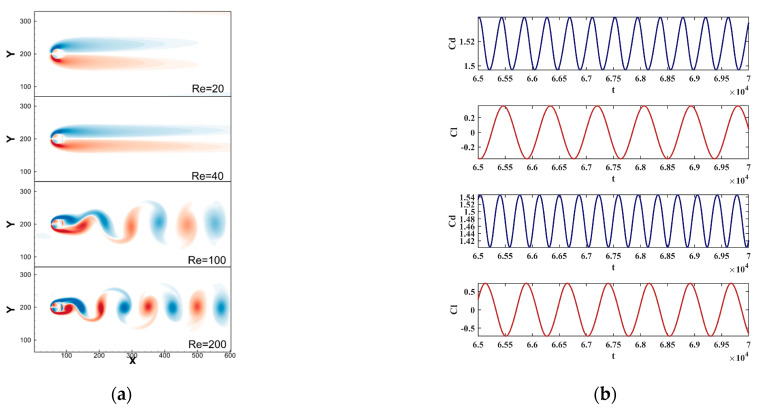
The results of different Reynolds numbers: (**a**) the vortex contours; and (**b**) the curves of lift coefficient Cl and drag coefficient Cd with Re = 100 and Re = 200.

**Figure 6:**
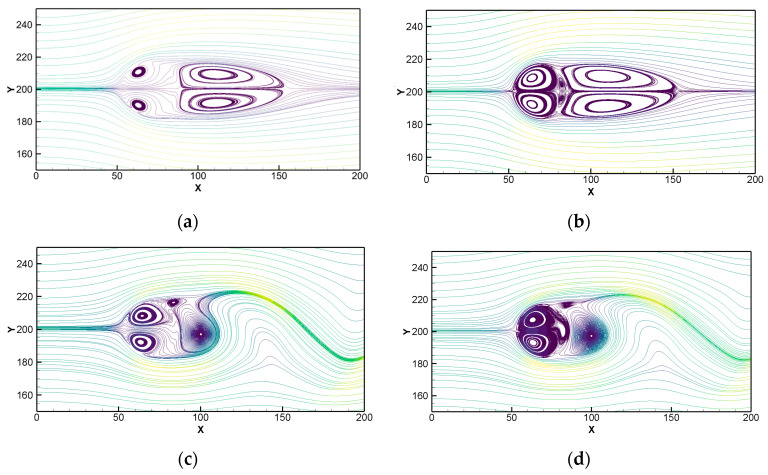
Comparison of the effect of matrix correction on the streamline penetration phenomenon at different Reynolds numbers: (**a**) Re = 40 without correction; (**b**) Re = 40 with correction; (**c**) Re = 200 without correction; and (**d**) Re = 200 with correction.

Figure 5a shows the vortex contours under four Reynolds numbers. It can be seen that they are basically consistent with the experimental phenomenon. When Re > 47, the flow separated and the unsteady flow phenomenon occurred. The Karman vortex street phenomenon was clearly visible at Re = 100 and Re = 200 and the vortex separation frequency increased with the increase of Reynolds number. Figure 5b shows two sets of drag coefficient curves and two sets of lift coefficient curves for Re = 100 and Re = 200 which correspond to the vortex contours of the same Reynolds number in Figure 6a. We see that as the Reynolds number increased, the vortex separation was obvious, which is reflected in the increase of the period numbers of the lift coefficient within a limited time. Its specific values are explained in Table 2. Figure 5a,b show that this method is accurate and effective in simulating unsteady flow.

Figure 6 shows the streamline diagrams of Section 4.2 under Re = 100 and Re = 200. In the case of Re = 40 (steady flow), as shown in Figure 6a without correction, there occurred the penetration of some streamlines inside the cylinder, which also occur in the previous explicit method and the conservation of mass cannot be guaranteed in this region. After the calculation of the modified Equation (30), the streamline became clear and no longer penetrated, which ensured the conservation of mass and satisfied the no-slip boundary condition. The same successful correction effect can be obtained from the streamline comparison of Figure 6c,d; that is to say, the streamline correction scheme proposed in this paper is applicable to unsteady flow.

### 4.3. Free Oscillation of the Flapping Foil NACA0012

The oscillation analysis of airfoil is an important subject of airfoil aerodynamic characteristics. The relevant studies are the prime motivators for the new technology in this regard, such as marine, aerial, and energy territory [64]. Using numerical methods to simulate and analyze relevant experimental conditions, such as wake state, has become an effective research method at present [65].

In this case, the two-dimensional airfoil NACA0012 was used and the chord length of the airfoil was *L*. The aerodynamic center $r_{c}$ was fixed, which was located at the center line of the foil with the distance $L_{c}=0.446L$ from the leading edge. Therefore, the airfoil is a movable, rigid body with one degree of freedom; the same settings can be seen in ref. [40]. The airfoil rotationally oscillated about the fixed point $r_{c}$. The dynamic equation of the airfoil was an ordinary differential equation (ODE).

(42) $$\int_{L}F_{f}\times \left(X-r_{c}\right)\cdot e_{z}d_{s}=I_{s}\frac{\partial^{2}\varphi }{\partial t^{2}}-C_{s}\frac{\partial \varphi }{\partial t}+K_{s}\varphi$$

where φ is the angle between the chord length and the horizontal direction, $I_{s}$ is the moment of inertia, $C_{s}$ is the damping coefficient, and $K_{s}$ is the stiffness coefficient.

The numerical domain was a rectangular domain $\Omega =\left[0,360\Delta x\right]\times \left[0,1200\Delta x\right]$ and the lattice length was Δx=1. The airfoil chord length was L=38Δx and the number of the Lagrangian grids was 72. The left side was the inlet and the inlet velocity was $U_{\infty }=0.05$. The right side was the outlet and the velocity gradient was 0. The four boundaries were all processed in the non-equilibrium scheme. The Reynolds number was Re=U∞Lν and the initial angle $\varphi_{0}=0$. Lift and drag coefficients of the airfoil were calculated using Equations (39) and (40). The ODE solution of the foil was solved by the conventional difference method and coupled with IB-LBM for the final solution. Four Reynolds numbers Re = 200, 500, 1000, and 2000 were used for numerical simulation.

**Figure 7:**
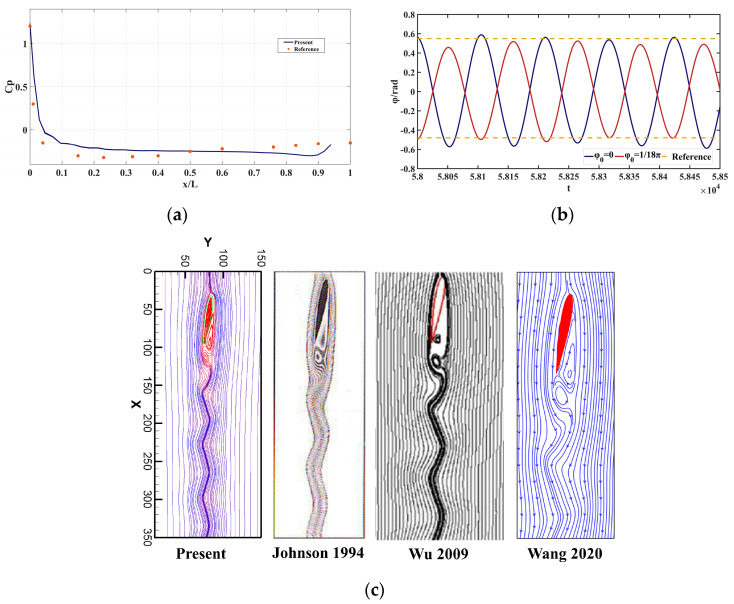
Comparison of the results of other papers: (**a**) pressure coefficient curve (Re = 500); (**b**) amplitude curve (Re = 1000); and (**c**) streamline (Re = 1000) [35,48,67].

Figure 7a shows the pressure coefficient curve along the edge of the airfoil when Re = 500, compared with ref. [66]. The pressure coefficient dropped rapidly from the center of the leading edge to a negative value and then approached 0, which also conforms to the changing characteristics of the NACA0012 airfoil standard pressure curve. Figure 7b shows a comparison of the periodic rotation angle curves of the airfoil when Re = 1000. It can be seen that the maximum angle was comparable to the vibration amplitude of ref. [40], and the maximum angle was about 0.6 rad. Similar to ref. [40], when changing the initial angle, the maximum rotation angle changed. Excessive maximum rotation angle in this case caused the program code to crash. Figure 7c shows the streamline comparison at ¼ T when Re = 1000 and the attack angle was 0.175 rad. In the simulation in this paper, the streamline had no penetration phenomenon. Compared with [35,48,67], the streamline results in this paper were similar to those in ref. [67]. The Strouhal number given in this paper was 0.855, which is similar to the 0.86 used in the literature [67].

**Figure 8:**
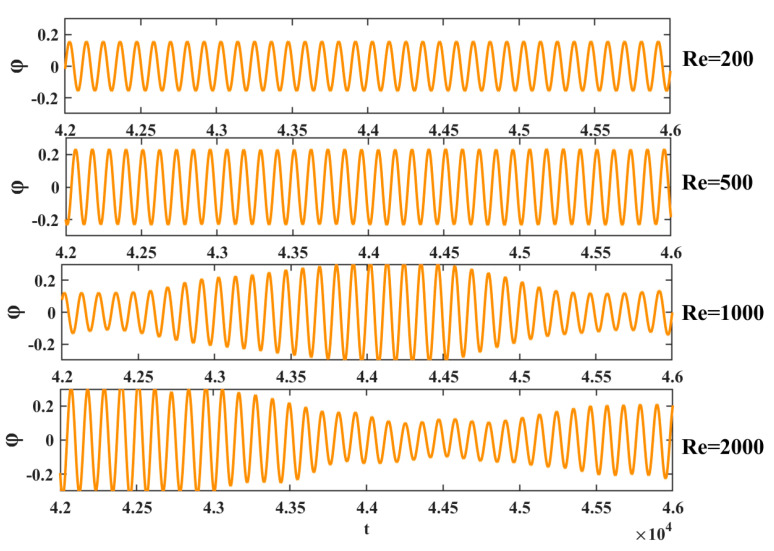
Rotation angle curves at four Reynolds numbers.

Figure 8 shows a comparison of the rotation angles at four Reynolds numbers. We see that the maximum rotation angle increased with the increase of the Reynolds number. When the rotation angle was too large, that is, the Reynolds number exceeded 4000, the numerical simulation failed. When Re = 200 and 500, the change of rotation angle was regular, and the rotation maximum amplitude was basically unchanged. However, when Re = 1000 and 2000, the rotation range increased and is related to the flow instability at a larger Reynolds number.

**Figure 9:**
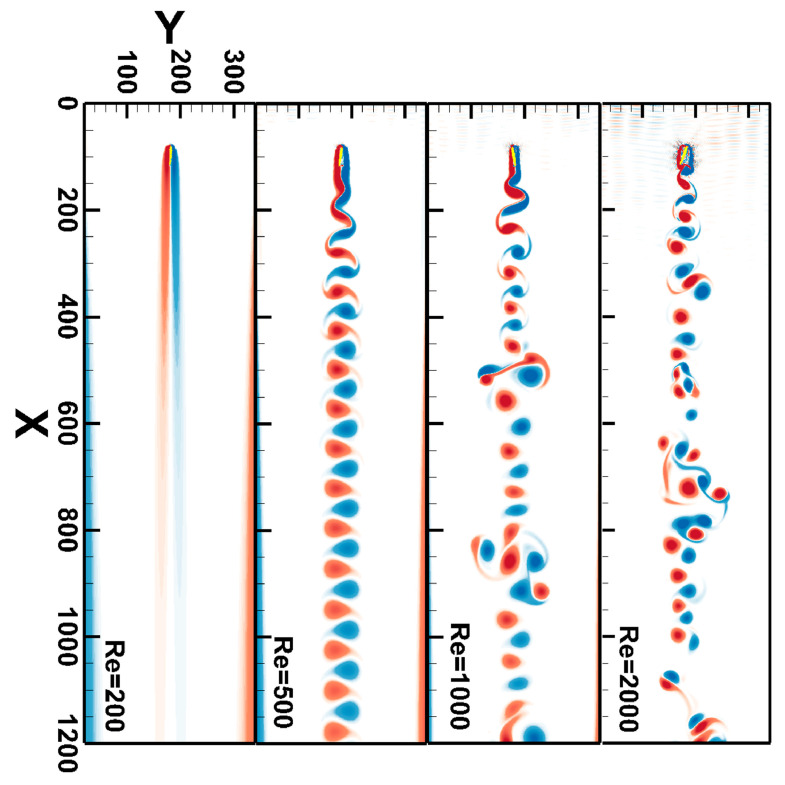
The vortex contours at four Reynolds numbers.

Figure 9 shows a comparison of the vortex contours of at four Reynolds numbers. It can be seen that when Re = 200, the flow was a laminar flow; when Re = 500, the flow became an unsteady flow, presenting a regular Karman vortex street; when Re = 1000, the downstream vortex shedding became unstable and presented a complex wake shape. Compared with Re = 1000, the unstable vortex shedding occurred earlier at Re = 2000, and this instability was more serious in the middle and later stages of the wake. When Re = 1000, 2000, we found that at the vertical line of the wake with the same horizontal position, vortex eyes appeared two to three times. This unstable vortex shedding is a common phenomenon and can be simulated by improving the model to realize the dual Karman vortex street [65].

### 4.4. A Flow Past Free Deformation of Cylinders

The deformation of the cylinders with flow around them constitutes an abstract model of practical engineering. In the numerical model of porous media, the skeleton model is often abstracted as cylinders around flow, so the research on the pore deformation of porous media can be transformed into the deformation of the cylinder group [68]. Additionally, changing the geometric parameters and solid force model, the cylinder deformation can provide a reference model in biology, such as in the deformation calculation of red blood cells [69].

The general deformation equation of an enclosed body is expressed by

(43) $$M\frac{\partial^{2}X}{\partial t^{2}}=F_{fluid}+F_{inner}=F_{fluid}+F_{T}+F_{N}$$

where M is the mass ratio, $F_{fluid}$ is the external force of the fluid acting on the solid, $F_{T}$ is the internal force in the tangential direction, and $F_{N}$ is the internal force in the normal direction. For $F_{T}$, $F_{N}$, this paper provides a force model that is proportional to the deformation which satisfies Newton’s law, and the sum of internal forces at all points is 0.

Adding the stretch coefficient $K_{s}$ and the bending coefficient $K_{b}$ and using the finite difference method, the specific deformation difference equation can be obtained by

(44) $$M\frac{X_{n}^{t+1}-2X_{n}^{t}+X_{n}^{t-1}}{\partial t^{2}}=F\left(X_{n}^{t},t\right)+F_{T}^{n}+F_{N}^{n}$$

Then,

(45) $$F_{T}^{n}\left(X_{n}^{t},t\right)=K_{s}\cdot \overset{n}{\underset{k=n-1}{\sum }}A_{k}\left(\Delta s_{k}^{t}-\Delta s_{k}^{0}\right)\tau_{k}^{t}$$

(46) $$F_{N}^{n}\left(X_{n}^{t},t\right)=K_{b}\cdot \overset{n+1}{\underset{k=n-1}{\sum }}B_{k}\left(\Delta \varphi_{k}^{t}-\Delta \varphi_{k}^{0}\right)N_{k}^{t}$$

where $\tau_{n}^{t}=\frac{X_{n+1}^{t}-X_{n}^{t}}{\Delta s}$ is the unit tangent vector and $N_{n}^{t}$ is the unit normal vector orthogonal to the vector $\left(X_{n+1}^{t}-X_{n-1}^{t}\right)$. The angle Δφ can be obtained from the relevant vectors.

Mark $X_{a,b}=X_{a}^{t}-X_{b}^{t}$; then, the coefficients $A_{k}$ and $B_{k}$ can be expressed by

(47) $$A_{k}=\left\{\begin{array}{l} 1,\;\;\;\;\;k=n\\ -1,\;k=n-1 \end{array}\right.$$

(48) $$B_{k}=\left\{\begin{array}{l} \frac{\left|X_{n,n-2}-X_{n,n-1}\right|}{\left(\left|X_{n,n-2}-X_{n-1,n-2}\right|+\left|X_{n,n-2}-X_{n,n-1}\right|\right)},k=n-1\\ 1,\;k=n\\ \frac{\left|X_{n+2,n}-X_{n+1,n}\right|}{\left(\left|X_{n+2,n}-X_{n+2,n+1}\right|+\left|X_{n+2,n}-X_{n+1,n}\right|\right)},k=n+1 \end{array}\right.$$

**Figure 10:**
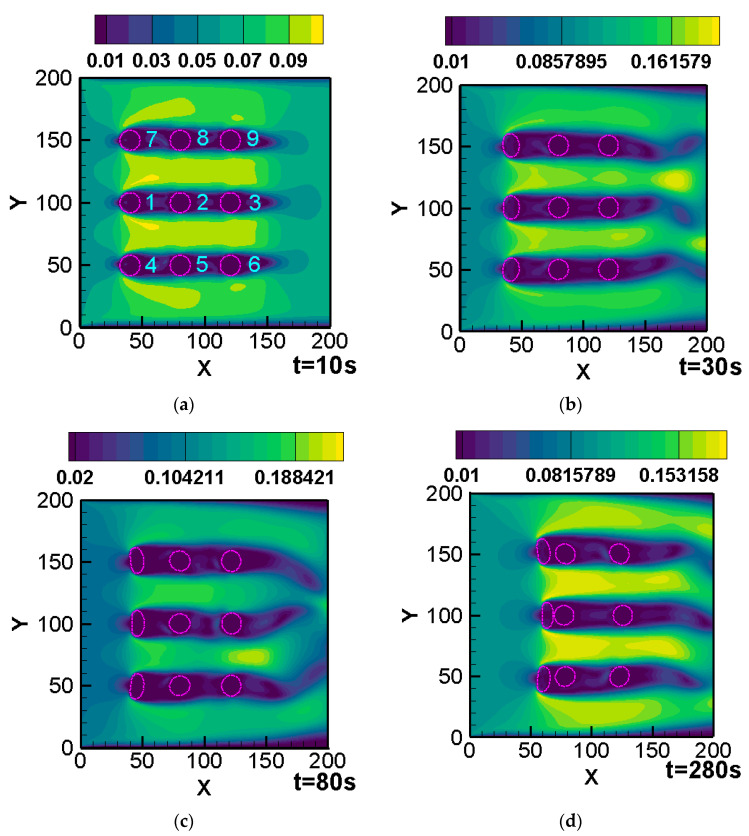
Deformation and velocity diagrams at four times: (**a**) 10 s; (**b**) 30 s; (**c**) 80 s; and (**d**) 280 s.

The computational domain was a rectangular area $\Omega =\left[0,2L\right]\times \left[0,L\right]$, L=200Δx, the grid length was Δx=1, and the time step was $\Delta t=10^{-3}s$. The left side was the inlet and the inlet velocity was $U_{\infty }=0.1$. The right side was the outlet and the velocity gradient was 0. The four boundaries were processed as the non-equilibrium scheme. Choosing deformable 2D cylinders, the diameter of each cylinder was 16Δx and the number of Lagrangian points was 36. The center of the first cylinder was $\left(40\Delta x,50\Delta x\right)$; a cylinder was arranged every 40Δx in the X direction and every 50Δx in the Y direction (see Figure 10a for specific labels of nine cylinders). The Reynolds number was Re=400, the maximum time steps setting was 240,000, and the calculation stopped if cylinders collided.

Figure 10 shows the velocity contour of flow field and deformation process of nine cylinders. The numerical domain was selected as 200Δx×200Δx. Comparing Figure 10a–d, it can be seen that the degrees of deformation and moving speeds of the cylinders (1,4,7) on the first column were greater than those of the second column, and similarly, the deformation and speed of the second column were greater than those of the third column. It can be seen that the cylinders on both sides (7,8,9 and 4,5,6) basically changed symmetrically, and the degree of deformation was smaller than that of the middle cylinder. Looking at the cylinders on the first column, cylinder 1 approached the rear cylinder earlier than cylinders 4 and 7. The velocity contours of the near-wake region behind the cylinder show that the attenuation of the wake velocity is the main reason for the different degrees of deformation.

**Figure 11:**
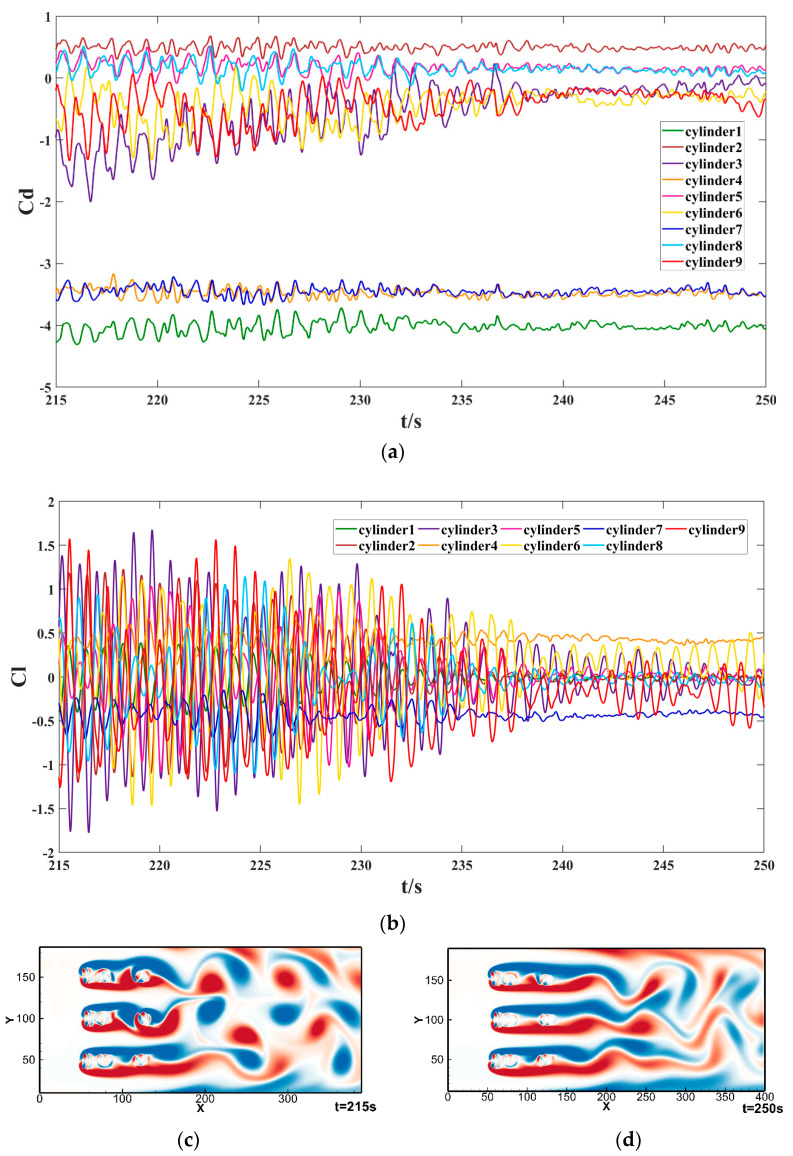
Diagram for deformation from 215 s to 250 s: (**a**) drag coefficient curve; (**b**) lift coefficient curve; (**c**) vortex contours at 215 s; and (**d**) vortex contours at 255 s.

Figure 11a,b are the lift and drag coefficient curves with time from 215 s to 250 s. It can be seen that the overall drag coefficient and lift coefficient decreased during this time period, especially for the third column (3, 6, 9). Due to geometric deformation, we think that the degree of attenuation in the near-wake region increased, which resulted in less drag force and thus less flow separation. The attenuation of the lift coefficient curve Figure 11b means the attenuation of the degree of flow separation. These inferences can be reflected from the vortex contours in Figure 11c,d and the flow separation of Figure 11c is greater than that of Figure 11d.

## 5. Conclusions

We summarized the content of the article and divided it into five points, as follows:

(1) We first obtained an explicit force with a simple form by assuming the existing LBM force model on IB in Equation (20). At present, the two most widely used explicit forces are a direct force proposed by Feng [33] and a bounce-back force proposed by Niu [44]. Feng’s direct force is similar to the stress integration method in LBM, and usually needs to calculate a complex stress tensor as in Equation (15); Niu’s bounce-back force is a combination of the bounce-back scheme and the momentum exchange method, and requires two steps: first, obtain the bounce-back distribution function (see [44] and a half-way bounce-back scheme optimized by Wang 2020 [48]), and then apply the momentum exchange method to obtain the interface force as in Equation (16). In contrast, our explicit force based on interpolating the distribution function and imposing the force model on the IB boundary can be realized in only one step, and the form is simple. Numerical experiments proved our force method and assumptions are accurate and effective;
(2) Then, we obtained the total explicit correction force scheme in Equation (30) by the correction matrix, which is an explicit scheme, only needing to add a matrix to Equation (20). The correction matrix T is only determined by the geometric position updated at time t−1, and the implicit scheme needs to solve the equation at time t. From the results of the clear streamline in Figure 6, the present scheme can well guarantee the local mass conservation, that is, almost satisfy the no-slip boundary condition, especially for the unsteady flow in Figure 6d and the large curvature boundary condition in Figure 7c. The correction matrix can be obtained by the LU decomposition method or some iterative methods;
(3) The proposed explicit correction force scheme has two modes for the user to choose: the first mode is Equation (11) and the second mode is Equation (11) and Equation (12) to obtain the interface force. When the user conducts a large number of grids or three-dimensional conditions for numerical simulation, the first mode can be selected to ensure acceptable simulation time. For medium-scale simulation, we recommend the full explicit correction force scheme to ensure clear results at the interface;
(4) For the accuracy order, we have the same order as the general IBM or IB-LBM, that is, the first-order accuracy order in Table 1. However, compared with the IBM by N-S solver, LBM has an excellent parallel strategy and local grid refinement strategy, which helps obtaining numerical simulation results with smaller errors;
(5) The proposed scheme performed well on complex boundaries such as moving boundaries in Section 4.3 and flexible boundaries in Section 4.4. Although good simulation results can be achieved on flexible boundaries by improving traditional implicit methods, explicit methods have traditional advantages in simulating complex boundaries. The proposed reference models in Section 4.3 and Section 4.4 have a background in the relevant discipline or engineering, and researchers can improve the proposed reference model to achieve good simulation results in specific cases.

## Figures and Tables

**Table 1:** The $L_{2}$ norm, the infinite norm of the error, and the convergence orders.

Mesh	$\left\|u_{n}-u_{a}\right\|$	Rate	${\left\|u_{n}-u_{a}\right\|}_{\infty }$	Rate
20×20	0.0797334		0.0858161	
40×40	0.0413924	0.9458183	0.0429297	0.9992722
80×80	0.0207219	0.9982096	0.0211221	1.0232228
160×160	0.00960611	1.1091321	0.00971167	1.1209620

**Table 2:** Comparison of the results on lift coefficient, drag coefficient, and Strohal number.

Parameter	Reference	Re = 200	Re = 100	Re = 40	Re = 20
$C_{D}$	Tritton [62]			1.48	2.22
	Calhoun [63]	1.17	1.33	1.62	2.19
	Hu [45]	1.394	1.418	1.660	2.213
	This study	1.471	1.518	1.755	2.335
$C_{L}$	Wu [35]		0.344		
	Calhoun [63]	0.67	0.298		
	Hu [45]	0.712	0.367		
	This study	0.711	0.3609		
St	Wu [35]	0.197	0.163		
	Calhoun [63]	0.202	0.175		
	Hu [45]	0.195	0.166		
	This study	0.216	0.185		

## Data Availability

Not applicable.
